# Function of histamine-driven cancer-associated fibroblast and hepatocyte growth factor in the progression of cholangiocarcinoma

**DOI:** 10.1093/gastro/goaf090

**Published:** 2025-10-01

**Authors:** Xin Wang, Guoli Sheng, Zhangdi Yan, Anda Shi, Zengli Liu, Yongchang Tang, Guangzhen Li, Zongli Zhang

**Affiliations:** Department of General Surgery, Qilu Hospital, Cheeloo College of Medicine, Shandong University, Jinan, Shandong, P. R. China; Department of General Surgery, Qilu Hospital, Cheeloo College of Medicine, Shandong University, Jinan, Shandong, P. R. China; Department of General Surgery, Qilu Hospital, Cheeloo College of Medicine, Shandong University, Jinan, Shandong, P. R. China; Department of General Surgery, Qilu Hospital, Cheeloo College of Medicine, Shandong University, Jinan, Shandong, P. R. China; Department of General Surgery, Qilu Hospital, Cheeloo College of Medicine, Shandong University, Jinan, Shandong, P. R. China; Department of General Surgery, Qilu Hospital (Qingdao), Cheeloo College of Medicine, Shandong University, Qingdao, Shandong, P. R. China; Department of General Surgery, Qilu Hospital, Cheeloo College of Medicine, Shandong University, Jinan, Shandong, P. R. China; Department of General Surgery, Qilu Hospital, Cheeloo College of Medicine, Shandong University, Jinan, Shandong, P. R. China; Department of General Surgery, Qilu Hospital, Cheeloo College of Medicine, Shandong University, Jinan, Shandong, P. R. China

**Keywords:** histamine, cancer-associated fibroblast, hepatocyte growth factor, cholangiocarcinoma, tumor microenvironment, scRNA-seq

## Abstract

**Background:**

Here, we aimed to identify the impact of the histamine (HA)–histamine receptor H2 (HRH2) signaling pathway on stimulating the secretion of hepatocyte growth factor (HGF) by cancer-associated fibroblasts (CAFs), as well as elucidating the mechanisms through which HGF promotes the progression of cholangiocarcinoma. In addition, our study has identified novel targets and investigated the potential use of Cimetidine and Capmatinib in cholangiocarcinoma.

**Methods:**

Single-cell RNA sequencing revealed a noteworthy correlation between the expression of *HRH2* and *HGF* in CAFs. HA was able to promote the transcription of HGF through the upregulation of the transcription factor hypoxia inducible factor 1 subunit alpha (HIF-1α), which was revealed by using *in vitro*/*vivo* experiments. That HGF promotes the progression of cholangiocarcinoma was identified by using orthotopic models and *in vitro* experiments.

**Results:**

HRH2 and HGF were primarily expressed in CAFs within the tumor microenvironment of cholangiocarcinoma. HA sourced from mast cells could bind to the HRH2 receptor on CAFs, consequently upregulating HIF-1α and subsequently enhancing the transcription and secretion levels of HGF. HGF upregulates the phosphorylation of FOS-like 1 (FOSL1) within cholangiocarcinoma cells, promoting the expression of matrix metallopeptidase 10 (MMP10), and consequently enhancing the invasive and migratory abilities of cholangiocarcinoma cells.

**Conclusions:**

The HA–HRH2 signaling pathway mediates the proliferation and secretion of HGF in CAFs. HIF-1α and FOSL1 played crucial roles in driving the proliferation, invasion, and migration of cholangiocarcinoma cells within the tumor microenvironment, orchestrated by CAFs. Furthermore, this study has provided theoretical support for the application of the HA receptor HRH2 inhibitor, Cimetidine, and the HGF receptor cellular mesenchymal–epithelial transition factor (c-MET) inhibitor, Capmatinib, in biliary tract tumors.

## Introduction

Cholangiocarcinoma (CCA) is a highly malignant cancer arising from the bile duct and can be classified into three subtypes, based on its anatomical location, as intrahepatic CCA (iCCA), perihilar CCA (pCCA), and distal CCA (dCCA) [1]. Patients have a poor prognosis due to the difficulty in early diagnosis and the limited effectiveness of adjuvant therapies such as radiation and chemotherapy [2]. By the time most patients with CCA seek medical attention, the window for surgical intervention has typically closed [3]. Even when patients meet the criteria for curative resection, the 5-year overall survival rate after radical resection for CCA remains at <20% [4]. Identifying high-risk patients for individualized treatments based on molecular characteristics remains challenging for CCA.

Hepatocyte growth factor (HGF) was widely recognized as a pleiotropic substance with mitogenic, motogenic, and morphogenic activities [5]. Studies have shown that moderate concentrations of HGF can stimulate the proliferation of iCCA cells *in vitro* [6–8]. Currently, there is a widespread belief that HGF originates from intrahepatic normal or cancer-associated fibroblasts (CAFs) [8]. Therefore, comprehending the mechanisms that trigger the secretion of HGF in CAFs is crucial within the tumor microenvironment of CCA.

The tumor microenvironment plays a pivotal role in the onset and advancement of CCA [9] and research on the mechanisms of CAFs in the tumor microenvironment of CCA is limited. Our prior investigations have revealed that histamine (HA), originating from mast cells (MCs), can promote CAFs to release HGF, consequently amplifying the proliferative capacity of CCA cells [7, 10]. However, the mechanisms by which HA promotes HGF release in CAFs and the mechanisms by which HGF enhances the proliferation, invasion, and migration capabilities of CCA cells are not fully revealed.

HA exerts diverse functions by interacting with its receptors, histamine receptor H1/2/3/4 (HRH1/2/3/4), each of which exhibits tissue-specific expression patterns [11]. In our study, we have identified that HRH2 and HGF were mainly expressed in CAFs within the CCA tumor microenvironment. Also, HA–HRH2 signaling could upregulate the transcription factor hypoxia inducible factor 1 subunit alpha (HIF-1α), thereby enhancing the transcription levels of HGF subsequently. Furthermore, HGF could upregulate the phosphorylation of FOS-like 1 (FOSL1) within CCA cells, leading to the expression of matrix metallopeptidase 10 (MMP10), thus enhancing the invasive and migratory abilities of CCA cells.

Ultimately, we have elucidated the secretion mechanism of HGF in CAFs mediated by the HA–HRH2 signaling pathway, as well as the signaling pathways and molecular mechanisms through which HGF promotes the progression of CCA with a series of *in vitro*/*vivo* experiments and a clinical cohort.

## Materials and methods

### CCA patients and ethics

A retrospective cohort and follow-up main cohort included 291 patients diagnosed with CCA and undergoing surgical resection at Qilu Hospital of Shandong University from 2013 to 2024, comprising 58 patients with iCCA, 113 patients with pCCA, and 120 patients with dCCA. The criteria for selecting these patients into the cohort were as follows: (i) patients who underwent radical resection with clear surgical margins; (ii) patients with formalin-fixed tumor tissue, complete follow-up, and medical records; (iii) patients with a postoperative survival time of >1 month; (iv) patients with no history of other malignant tumors. Tumors were categorized and staged according to the 8th edition of the AJCC/UICC TNM classification system and patient prognosis was monitored through follow-up. The study received approval from the Ethics Committee of Qilu Hospital of Shandong University.

### Tissue microarray and immunohistochemistry

Tissue microarray was constructed by using paraffin-embedded tissues as previously reported. The tumor area was first identified after hematoxylin and eosin staining by a senior pathologist (X.Y.). Core biopsies measuring 1.5 mm in diameter were taken from each sample and arranged into tissue microarray slides.

Deparaffinization of the paraffin-embedded tissue sections was followed by antigen retrieval in 10 mmol/L of sodium citrate buffer (pH 6.0). The tissue sections were then incubated overnight at 4°C with primary antibodies cellular mesenchymal–epithelial transition factor (c-MET; 1:200), MMP10 (1:200), Heat shock protein 60 (Hsp60; 1:200), and β-catenin (1:100). Subsequently, the sections were incubated with the appropriate secondary antibodies (Zsbio, Beijing, China) at room temperature for 30 min. The slides were then incubated with 3,3-diaminobenzidine solution (Zsbio) coupled with horseradish peroxidase-streptavidin.

Quant Center software was used to analyse the immunohistochemistry (IHC) staining results by calculating the synthetic score of the staining intensity and the area of each staining. The IHC score was calculated by using the formula: (percentage of weakly stained cells × 1) + (percentage of moderately stained cells × 2) + (percentage of strongly stained cells × 3). Based on the expression levels of the target proteins, patients in the cohort were divided into different subsets. The cutoff values for the IHC scores were determined by using receiver-operating characteristic curve analysis.

### Cell lines and agents

The human intrahepatic CCA cell line RBE, the extrahepatic CCA cell line QBC-939, the human normal biliary epithelial cell line HIBEpiC, the human MC line HMC-1, and the mouse intrahepatic CCA cell line LD-1 were all purchased from the Cell Bank of the Chinese Academy of Sciences (Shanghai, China). LD-1 is a murine iCCA cell line widely utilized in both *in vivo* and *in vitro* studies. It displays characteristic features of CCA and has been thoroughly characterized for its tumorigenic capacity and molecular relevance to CCA. RBE and LD-1 were cultured in RPMI 1640 medium (Thermo Fisher Scientific, Waltham, MA, USA), while QBC-939, HIBEpiC, and HMC-1 were cultured in DMEM (Thermo Fisher Scientific). Both types of media were supplemented with 10% fetal bovine serum (Thermo Fisher Scientific), 100 U/mL of penicillin, and 100 μg/mL of streptomycin. All cells were cultured in an incubator at 37°C with 5% CO_2_. The information on reagents and antibodies is detailed in Supplementary Table S1.

### Primary CAFs isolation and culture

Fresh tissue samples from patients with CCA were collected during surgery and washed in high-concentration antibiotic phosphate-buffered saline (PBS) containing 1,000 U/mL of penicillin and 1,000 U/mL of streptomycin to remove blood from the tissue surface. The fat and connective tissue on the surface was stripped away and the tissue was repeatedly washed in PBS until the liquid was clear. The diseased tissue was then cut into pieces that measured 2–3 mm by using sterile scalpels. The tissue was placed in 4 mL of 0.25% trypsin-ethylenediaminetetraacetic acid (trypsin-EDTA) digestion solution and digested at 37°C and 200 rpm on a benchtop shaker for 30 min. The digestion was terminated with 8 mL of pre-warmed 37°C RPMI-1640 complete medium and the digestion product was filtered through a 70-μm strainer. We added 2 mL of complete medium (HXXFB-90011, Oricell, Shanghai, China) to each well of a six-well plate and transferred the tissue fragments to the culture dishes. The medium was changed every 48 h until fibroblasts extended from the digested tissue. After 30–40 days, the residual tissue was removed and the fibroblasts were trypsinized and passaged by using trypsin/EDTA (Solarbio, Beijing, China). CAFs were identified by using immunofluorescence staining for α-smooth muscle actin (α-SMA) and fibroblast activation protein (FAP), as shown in Supplementary Figure S2.

### RNA extraction and quantitative polymerase chain reaction

Total RNA was extracted from cells and tissues by using TRIzol reagent (Thermo Fisher). The RNA was then reverse-transcribed into complementary DNA (cDNA) by using a reverse transcriptase kit (TOYOBO, Japan). Real-time quantitative polymerase chain reaction (RT-qPCR) was performed by using SYBR Green Master Mix (Roche, USA) and the QuantStudio 5 PCR instrument (Thermo Fisher). The 2^−ΔΔCt^ method was used to calculate the relative expression, with GAPDH (glyceraldehyde 3-phosphate dehydrogenase) serving as an internal control. The primers used for qPCR are listed in Supplementary Table S2.

### Western blotting

Total protein from tissues and cells was extracted by using RIPA lysis buffer (Solarbio Science, Beijing, China) containing 1% PMSF (Beyotime, Shanghai, China) and 1% phosphatase inhibitor (Solarbio). Nuclear and cytoplasmic proteins were separated by using the Nuclear Protein Extraction Kit (Solarbio) according to the manufacturer’s instructions. The extracted protein solution was mixed with 5× sodium dodecyl sulfate–polyacrylamide gel electrophoresis (SDS-PAGE) loading buffer (Spark, China) and heated to denature the proteins. Protein samples were then subjected to electrophoresis on a 10%–12.5% SDS–PAGE gel and transferred to a polyvinylidene fluoride (PVDF) membrane (Millipore, Bedford, MA, USA). Then, the transferred proteins on the PVDF membrane were blocked with 5% bovine serum albumin (BSA) as the blocking solution and incubated with specific primary antibodies of HRH1 (1:1,000), HRH2 (1:1,000), HGF (1:1,000), HIF-1α (1:1,000), c-MET (1:1,000), phospho-c-MET (1:1,000), ERK1/2 (1:1,000), phospho-ERK1/2 (1:1,000), AKT (1:1,000), phospho-AKT (1:1,000), STAT3 (1:1,000), phospho-STAT3 (1:1,000), MMP9 (1:1,000), MMP10 (1:1,000), MMP13 (1:1,000), FOSL1(1:1,000), phospho-FOSL1(1:1,000), Histone H3 (1:1,000), α-SMA (1:1,000), FAP (1:1,000), or β-actin (1:1,000) at 4°C overnight, followed by incubation with secondary antibodies (1:10,000) for 1 h. Finally, the protein bands were developed by using enhanced chemiluminescence (Millipore) and analysed through ImageJ software.

### Transfection and transient cell lines

CAF, RBE, and 939 cells were cultured in six-well plates. Gene knockdown or overexpression in RBE, 939, and CAFs was achieved through siRNA and plasmid transfection. Cells were transfected by using Lipofectamine 3000 (Thermo Fisher) and the efficiency of knockdown and overexpression was verified by using Western blotting (WB) and real-time quantitative PCR. The target sequences for the siRNAs are provided in Supplementary Table S3.

### Transwell assays

A total of 5 × 10^6^ RBE cells or MMP10-silenced RBE cells, or 1 × 10^5^ QBC-939 cells or MMP10-silenced RBE cells were resuspended in 200 μL of serum-free medium and placed in the upper chambers of transwell inserts (8.0 μm pore size; Corning), either with or without Matrigel (diluted 1:6 with RPMI 1640 or DMEM; Corning). The lower chambers contained 600 μL of 10% serum-conditioned medium or supernatants derived from CAF/HGF-silenced CAFs. In the stimulation groups, the RBE and 939 cells were treated with Capmatinib (20 nM) for 6 h, then resuspended in 200 μL of serum-free medium containing the same concentration of the inhibitor. After 36 h of incubation, adherent cells were fixed with methanol and stained with 0.5% crystal violet (Beyotime) for 30 min. The transwell chambers were then washed three times with PBS, and non-migrating or non-invading cells were removed by using cotton swabs. Five random fields of view were photographed under a microscope at 200× magnification and the number of cells was counted by using ImageJ software [12, 13].

### Wound-healing assay

Cells were seeded in six-well plates at a density of 2 × 10^5^ cells per well and cultured at 37°C. After the cells had adhered, a wound was created in the cell monolayer by using a sterile pipette tip. The cells were then washed twice with cold PBS and the initial wound size was measured by using a microscope. Subsequently, the cells were cultured in serum-free medium for 24 h at 37°C, after which the wound size was measured again. The percentage of wound closure was calculated by using the formula: (1 − [final wound size/initial wound size]) × 100.

### Cell proliferation assay

Cell proliferation assays were conducted using the procedure provided by the Cell Counting Kit-8 (Dojindo, Kumamoto, Japan). Briefly, 3 × 10^3^ of tumor cells were cultured in 200 μL of complete medium with or without HGF (30 ng/mL) or in the supernatant of CAF/HGF-silenced CAFs. The cell mixture was then incubated in a 96-well plate for 0, 24, 48, 72, and 96 h. After incubation, 10 μL of CCK-8 was added to each well, followed by incubation at 37°C for 1 h. The absorbance value at 450 nm was detected by using a spectrophotometer (Molecular Devices Company, France).

### Immunofluorescence assay

A total of 5 × 10^4^ CAF cells were seeded in a 24-well plate containing standard coverslips and cultured for 24 h. After being washed three times with PBS, the cells were fixed with 4% paraformaldehyde for 20 min. The cell membranes were then permeabilized with 0.2% Triton X-100 for 10 min, followed by three PBS washes. The cells were incubated with 5% goat serum at room temperature for 1 h and then with specific primary antibodies (diluted in 5% serum) overnight. The following day, the primary antibodies were removed and the coverslips were washed three times with PBS. The cells were then incubated with goat anti-mouse Alexa Fluor 488 (Invitrogen, Waltham, MA, USA; Cat# A-21202) and goat anti-rabbit Alexa Fluor 594 (Invitrogen; Cat# A-21207) fluorescent secondary antibodies for 1 h and stained with 4′,6-diamidino-2-phenylindole (DAPI) to visualize the nuclei. After an anti-fade reagent was applied, the coverslips were mounted onto slides by using neutral resin. Images were captured by using a confocal microscope (Carl Zeiss, LM780) [14].

### Enzyme-linked immunosorbent assay

The concentration of human HGF in the supernatant of primary CAF cells was measured by using an HGF enzyme-linked immunosorbent assay (ELISA) kit. All procedures were performed according to the manufacturer’s instructions. The ELISA absorbance values were read at 450 nm by using a BIOTEK spectrophotometer (Vermont, USA).

### Flow cytometry

The levels of c-MET and p-c-MET in QBC-939 and RBE were measured by using flow cytometry (FCM). Briefly, cells were collected and permeabilized with 0.5% Triton for 10 min, then stained with primary antibodies at 4°C overnight. After three washes with PBS, the cells were incubated with Alexa Fluor 488 dye-conjugated secondary antibodies at room temperature for 3 h in the dark. The fluorescence intensity of the c-MET and p-c-MET was detected by using a flow cytometer and the data were analysed by using FlowJo software.

### Single-cell RNA sequencing

Single-cell RNA sequencing (scRNA-seq) was commissioned by LC-Bio Technologies (Hangzhou, China). Single-cell suspensions were prepared from three pCCAs, four dCCAs, and four tumor-adjacent bile duct tissues, and the cell concentration was adjusted to 700–1,200 cells/μL. The 10× Chromium system was used to capture 5,000 single cells. Subsequent cDNA amplification and library construction were performed. The libraries were sequenced on an Illumina NovaSeq 6000 sequencing system at a minimum depth of 20,000 reads per cell. Cell types were annotated by using cluster-specific genes and classic markers [14, 15].

To enhance the comprehensiveness of our analysis, we integrated the newly generated scRNA-seq data (GSE213452) with the publicly available iCCA scRNA-seq data (GSE138709). All scRNA-seq analyses in this study were conducted based on this integrated dataset.

### *In vivo in situ* model of intrahepatic CCA

Specific pathogen-free (SPF)-grade 8-week-old female C57BL/6 mice were purchased from Vital River (Beijing Vital River Laboratory Animal Technology Co., Ltd) and randomly divided into groups. After the mice were anesthetized by using a gas anesthesia machine and their abdomens were disinfected with alcohol, an incision was made along the midline from below the xiphoid process to open the skin and peritoneum, exposing the left liver lobe. Using an insulin needle, 20 μL containing 1 × 10^6^ LD-1 cells was injected. Mice with successful orthotopic models were given recombinant HGF (50 ng/kg/day) and Capmatinib (30 mg/kg/day). The intrahepatic tumor weight was measured after 4 weeks [16]. All of our animal studies were approved by the Ethics Committee of Qilu Hospital of Shandong University (approval no. KYLL-2020–201 and no. KYLL-2021– 624), respectively.

### RNA sequencing

The total mRNAs from LD-1 were stimulated with or without mouse-purified HGF (30 ng/mL) by using TRIzol reagent (Invitrogen). RNAs were sequenced by using the Illumina HiSeq 4000 platform (LC-BioTechnologies, Zhejiang, China). Clean reads were aligned to the reference genome in orientation mode by using TopHat software. Sequence reads were mapped to the reference genome by using HISAT2 software. Transcript abundances (read counts) were estimated by using StringTie. Normalization and differential expression analysis of mRNAs were performed by using the DESeq2 package in R4.3.2 [17, 18].

In addition to our own RNA-seq analysis, two publicly available datasets were used to support and validate our findings. GSE26566 is a microarray dataset that includes gene-expression profiles from 104 human CCA samples, covering both intrahepatic and extrahepatic subtypes, along with 59 matched adjacent non-tumor liver tissues. GSE 132305 is a bulk RNA-sequencing dataset comprising 182 tumor tissues and 38 adjacent normal bile duct tissues from patients with extrahepatic CCA.

### The caudal vein metastasis model

SPF-grade 8-week-old female C57BL/c nude mice were randomly divided into groups of four each. Each mouse was injected with 100 μL of stable high-expression EGFP RBE cells (MMP10 knockdown) at a concentration of 4 × 10^5^ cells via the tail vein, with or without recombinant HGF (50 ng/kg for 7 days) and Capmatinib (30 mg/kg/day for 14 days). Tumor metastasis was monitored by using a live imaging system (IVIS) spectrum and the radiance efficiency was measured by using the IVIS Imaging Systems software to quantify the tumor burden in the mice [19].

### For the splenic metastasis model

SPF 8-week-old female C57BL/c nude mice were randomly assigned, with six mice per group. The spleen was gently extracted from the abdominal cavity and 20 μL of Matrigel containing of 1 × 10^6^ RBE cells (MMP10 knockdown) were injected into the spleen by using an insulin needle. Following anesthesia and recovery, the mice were maintained under SPF conditions. Each group of mice received a normal diet with or without recombinant HGF (50 ng/kg for 7 days) and Capmatinib (30 mg/kg/day for 14 days). After 4 weeks of *in situ* injection, the mice were sacrificed. The weight and size of the spleen *in situ* tumor, as well as the number of nodules in the liver and lungs, were determined though hematoxylin and eosin staining and the immunohistochemical verification of human mitochondrial markers.

### Statistical analysis

Statistical analyses were conducted by using SPSS 26.0 (IBM Corp., Armonk, NY, USA) and GraphPad Prism 8.1 (GraphPad Software, San Diego, CA, USA) software. One-way analysis of variance (ANOVA), two-way ANOVA, or *t*-tests were employed to compare statistical differences between the groups. *P* values of <0.05 were considered significant.

## Results

### Expression profile of HRH receptors and HGF in CCA

**Figure 1.**
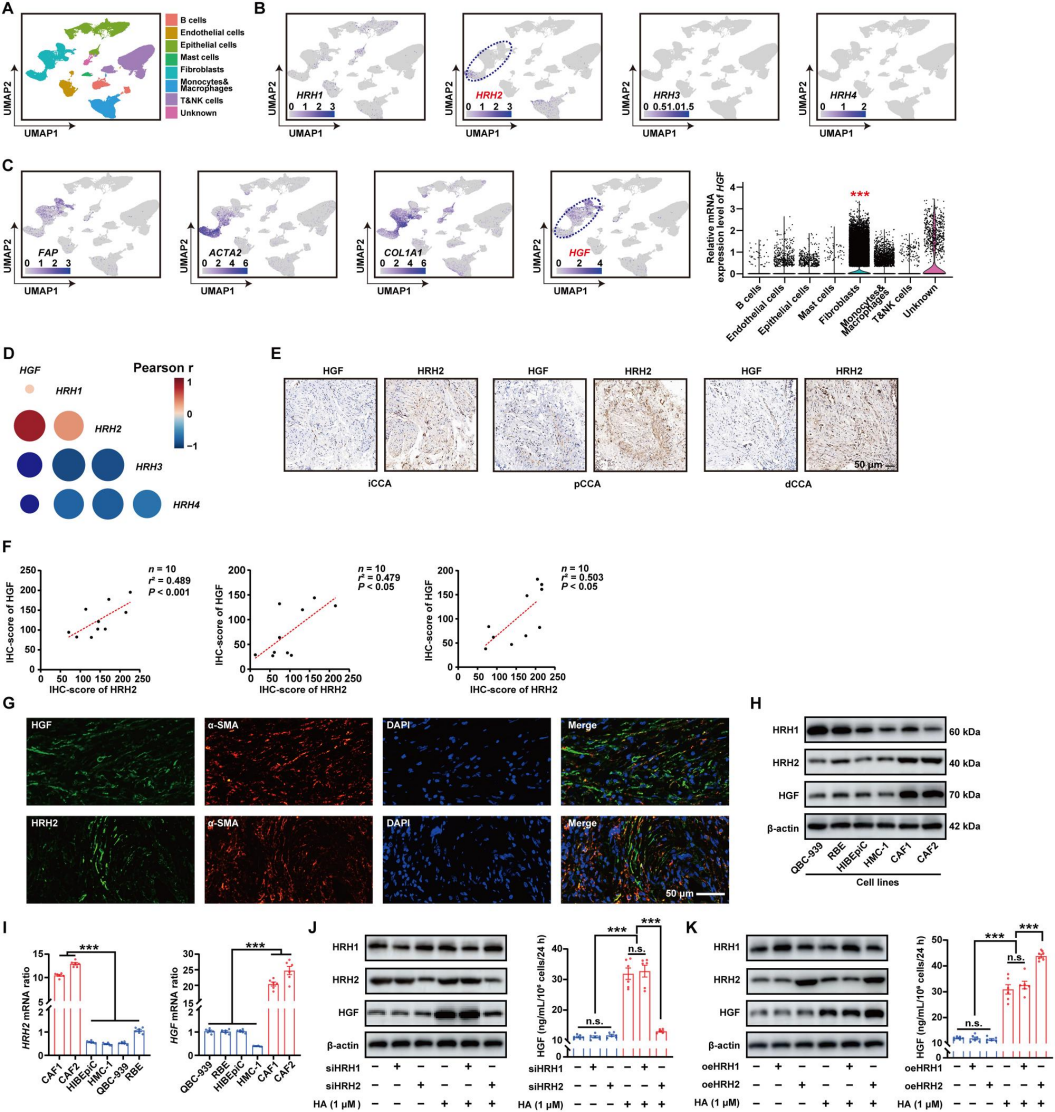
HA–HRH2 signaling pathway facilitates the secretion of HGF in CAFs. (A) scRNA-seq data from three pCCAs, four dCCAs, and four tumor-adjacent tissues that were integrated with previous data of iCCA. A total of 102,814 single cells were clustered into eight major clusters. (B, C) In uniform manifold approximation and projection (UMAP), *HRH2* and *HGF* were mainly upregulated in CAFs. Violin plots illustrate the relative mRNA expression level of *HGF* in various annotated cell types of the scRNA-seq dataset. *** represents *P* < 0.001 compared with other annotated cell types in (C). (D) According to scRNA-seq data, *HRH2* exhibits the strongest correlation with *HGF* expression in CAFs. (E) Representative images of HRH2 or HGF expression in iCCA (*n* = 10), pCCA (*n* = 10), and dCCA (*n* = 10) tumor tissues. Scale bars: 50 µm. (F) Correlation between HRH2 and HGF protein expression in iCCA, pCCA, and dCCA was examined. (G) α-SMA, HGF, and HRH2 in CCA tissues were detected through immunofluorescence. Scale bar: 50 µm. (H, I) Expressions of HRH1, HRH2, and HGF were detected in HIBEpiC, QBC-939, RBE, HMC-1, and primary CAFs through (H) WB and (I) qPCR. (J) Following separate knockdown of HRH1 and HRH2, CAFs were stimulated with 1 μM of HA for 24 h. The expression and secretion of HGF were detected by using WB (left) and ELISA (right). (K) Following separate overexpression of HRH1 and HRH2, CAFs were stimulated with 1 μM of HA for 24 h. The expression and secretion of HGF were detected by using WB (left) and ELISA (right). n.s. represents not significant. *, **, and *** represent *P* < 0.05, 0.01, and 0.001, respectively. Statistical significance was analysed by using one-way ANOVA (C, right; I, J, right; and K, right) or Pearson test (F). Data were from at least three independent experiments and are shown as mean ± standard error of the mean (SEM).

We performed scRNA-seq on samples from three pCCAs, four dCCAs, and four tumor-adjacent tissues (GSE213452) and integrated these data with previously published scRNA-seq data on iCCA (GSE138709) [20]. A total of 102,814 single cells were clustered into eight major clusters (Figure 1A). Our previous study demonstrated that MCs can secrete HA into the tumor microenvironment of CCA [7]. Also, HA receptors HRH1/2/3/4 exhibit tissue-specific expression and can activate distinct downstream signaling pathways by coupling with Gα proteins as a type of G protein-coupled receptor. In our scRNA-seq data of CCA, *HRH2* and *HGF* were mainly expressed in CAFs (Figure 1B and C), whereas *HRH3/4* were barely expressed in CCA. Furthermore, *HRH2* has shown the highest correlation with *HGF* expression in CAFs through scRNA-seq (Figure 1D) and this result was also validated by the CCA databases (GSE26566 and GSE132305) (Supplementary Figure S1). To verify the bioinformatic findings, we performed immunohistochemical staining for HGF and HRH2 on tumor tissues from iCCA (*n* = 10), pCCA (*n* = 10), and dCCA (*n* = 10), with subsequent correlation analysis showing a positive correlation between their expression (Figure 1E and F). Additionally, colocalization immunofluorescence assays further confirmed these findings (Figure 1G).

### HA–HRH2 signaling pathway facilitates the secretion of HGF in CAFs

To investigate the HGF expression in the CAFs, we isolated primary CAF cells from CCA tissue samples and identified them by detecting the biomarkers α-SMA and FAP through immunofluorescence (Supplementary Figure S2). With WB and qPCR, we validated that HRH2 and HGF were highly expressed in CAFs (Figure 1H and I). Following the modulation of HRH1 or HRH2 expression through either knockdown or overexpression separately, 1 μM of HA was used to stimulate the CAFs for 24 h. Ultimately, we demonstrated that the secretion of HGF in CAFs is mediated by the HA–HRH2 signaling pathway (Figure 1J and K).

### Expression profile of HIF-1α in CAFs

**Figure 2.**
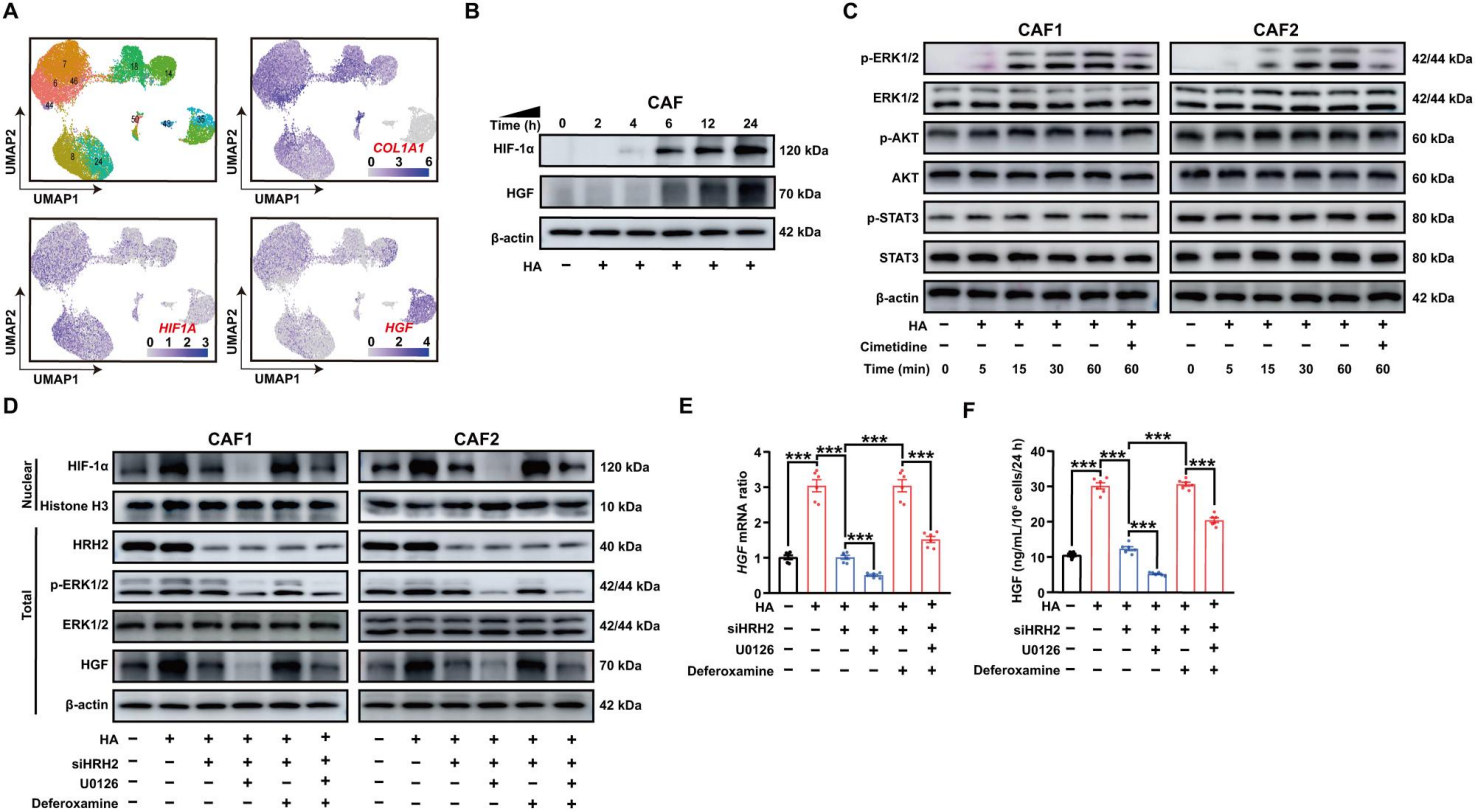
HIF-1α mediates the expression and secretion of HGF. (A) In UMAP, *HIF1A* and *HGF* were observed to be co-expressed in all subtypes of CAFs. (B, C) Time–effect correlation between HA (1 μM) stimulation and the expression of HIF-1α, HGF, and phosphorylation of ERK1/2, AKT, and STAT3 were evaluated by using WB in the presence/absence of Cimetidine (HRH2 inhibitor, 10 μM). (D) In the presence/absence of HA (1μM), U0126 (ERK inhibitor, 20 μM), Deferoxamine (HIF-1α agonist, 100 μM), or siRNA of HRH2, WB (D). (E, F) In the presence/absence of HA (1 μM), U0126 (20 μM), Deferoxamine (100 μM), or siRNA of HRH2, the mRNA expression level and secretion of HGF were detected by using (E) WB and (F) ELISA. n.s. represents not significant; *, **, and *** represent *P* < 0.05, 0.01, and 0.001, respectively. Data were analysed by using one-way ANOVA (E, F). Data were from at least three independent experiments and are shown as mean ± SEM.

In our scRNA-seq dataset, *HIF1A* and *HGF* were found to be co-expressed in all subtypes of CAFs and a consistent correlation was also observed (Figure 2A). Furthermore, a significant positive correlation between the expression levels of *HIF1A*, *HRH2*, and *HGF* was observed in the CCA database (GSE26566), as shown in Supplementary Figure S3.

### HA–HRH2 signaling pathway promotes the translocation of transcription factor HIF-1α into CAF nucleus

From WB, we observed the regulated expression of HIF-1α and HGF, as well as the activation of mitogen-activated protein kinase (MAPK) (extracellular signal regulated kinase (ERK)) phosphorylation within the HA–HRH2 signaling pathway (Figure 2B and C, and Supplementary Figure S4). To investigate the nuclear translocation of HIF-1α in CAFs mediated by the HA–HRH2 signaling pathway, we isolated proteins from CAFs and conducted nuclear component analysis. HA promotes the nuclear translocation of HIF-1α, whereas knockdown of HRH2 and inhibition of ERK phosphorylation with U0126 suppress this effect (Figure 2D). The HA–HRH2 signaling pathway ultimately enhances HIF-1α expression and facilitates its translocation into the nucleus. With qPCR and ELISA tests, we showed that HA significantly increased both the transcription and secretion of HGF (Figure 2E and F).

### HGF promotes the proliferation, invasion, and migration capabilities of CCA cells

**Figure 3.**
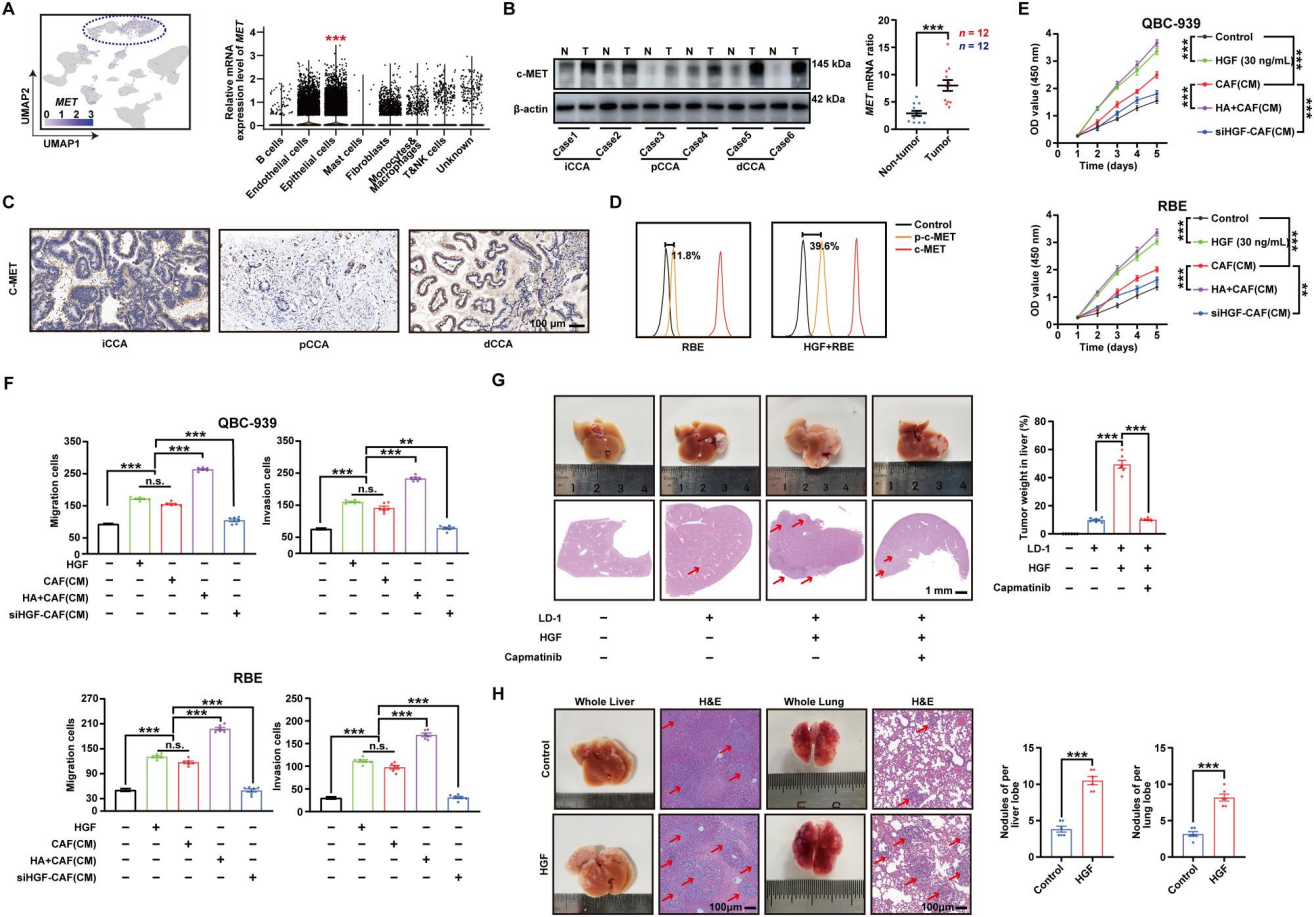
HGF promotes the proliferation, invasion, and migration capabilities of CCA cells. (A) Left: In UMAP, *MET* was mainly upregulated in tumor epithelial cells. Right: Violin plots illustrate the relative mRNA expression level of *MET* in various annotated cell types of the scRNA-seq dataset. *** represents *P* < 0.001 compared with other annotated cell types. (B) Expression of c-MET was detected in iCCA, pCCA, and dCCA tissues, as well as their paratumor tissues, by using WB (left) and qPCR (right). (C) Representative IHC Images of c-MET expression in iCCA, pCCA, and dCCA tumor tissues. Scale bars: 100 µm. (D) Phosphorylation of c-MET was detected under the stimulation of HGF by using FCM. (E, F) CCA cells were incubated in HGF (30 ng/mL) and CM from CAFs or HGF-silenced CAFs, which were stimulated with HA (1 μM). CCA cell proliferation was assessed by using (E) CCK8 assay and (F) transwell assay. (G) Left: iCCA orthotopic tumor models were established in wild-type mice by injecting mouse iCCA cells (LD-1). HGF (50 ng/kg/day, 7 days) was administered via intraperitoneal injection and the c-MET receptor inhibitor Capmatinib (30 mg/kg/day, 14 days) was given orally. Right: The ratio of tumor weight in the liver was measured. (H) Left: After immunocompromised nude mice were injected with the CCA cell line RBE, HGF was administered via intraperitoneal injection. Right: The number of nodules per lobe in the liver and lung was measured. n.s. represents not significant. *, **, and *** represent *P* < 0.05, 0.01, and 0.001 between the indicated groups. Statistical significance was analysed by using one-way ANOVA (F, G, right), two-way ANOVA (E), unpaired *t*-test (H, right), or paired *t*-test (B, right). Data were from at least three independent experiments and are shown as mean ± SEM.

In the CCA microenvironment, scRNA-seq analysis revealed a predominant expression of *MET* in tumor epithelial cells (Figure 3A and Supplementary Figure S5). By using WB and qPCR, we verified that c-MET exhibited significantly higher expression levels in CCA tissues compared with adjacent paratumoral tissues (Figure 3B). Immunofluorescence staining further demonstrated predominant localization of c-MET in tumor epithelia (Figure 3C). Following HGF stimulation, FCM analyses revealed upregulated phosphorylation of c-MET in the CCA cell line RBE (Figure 3D). Moreover, conditioned media (CM) from CAFs or HA-pretreated CAFs could promote CCA cell proliferation, invasion, and migration, while HGF knockdown blocked this effect (Figure 3E and F, and Supplementary Figure S6). To further validate our previous results, we planted mice CCA cells LD-1 into the liver to establish an orthotropic model. HGF was administered via intraperitoneal injection and the c-MET receptor inhibitor Capmatinib was given orally. The orthotropic model further validated that HGF promoted the proliferation of CCA cells *in vivo* (Figure 3G). Furthermore, after immunocompromised nude mice were injected with the CCA cell line RBE, HGF was administered via intraperitoneal injection. Examination of the liver and lung tissues of the mice revealed the critical roles of HGF in the distant metastasis process of CCA cells (Figure 3H).

### HGF promotes the upregulation of MMP10 expression in CCA cells

**Figure 4.**
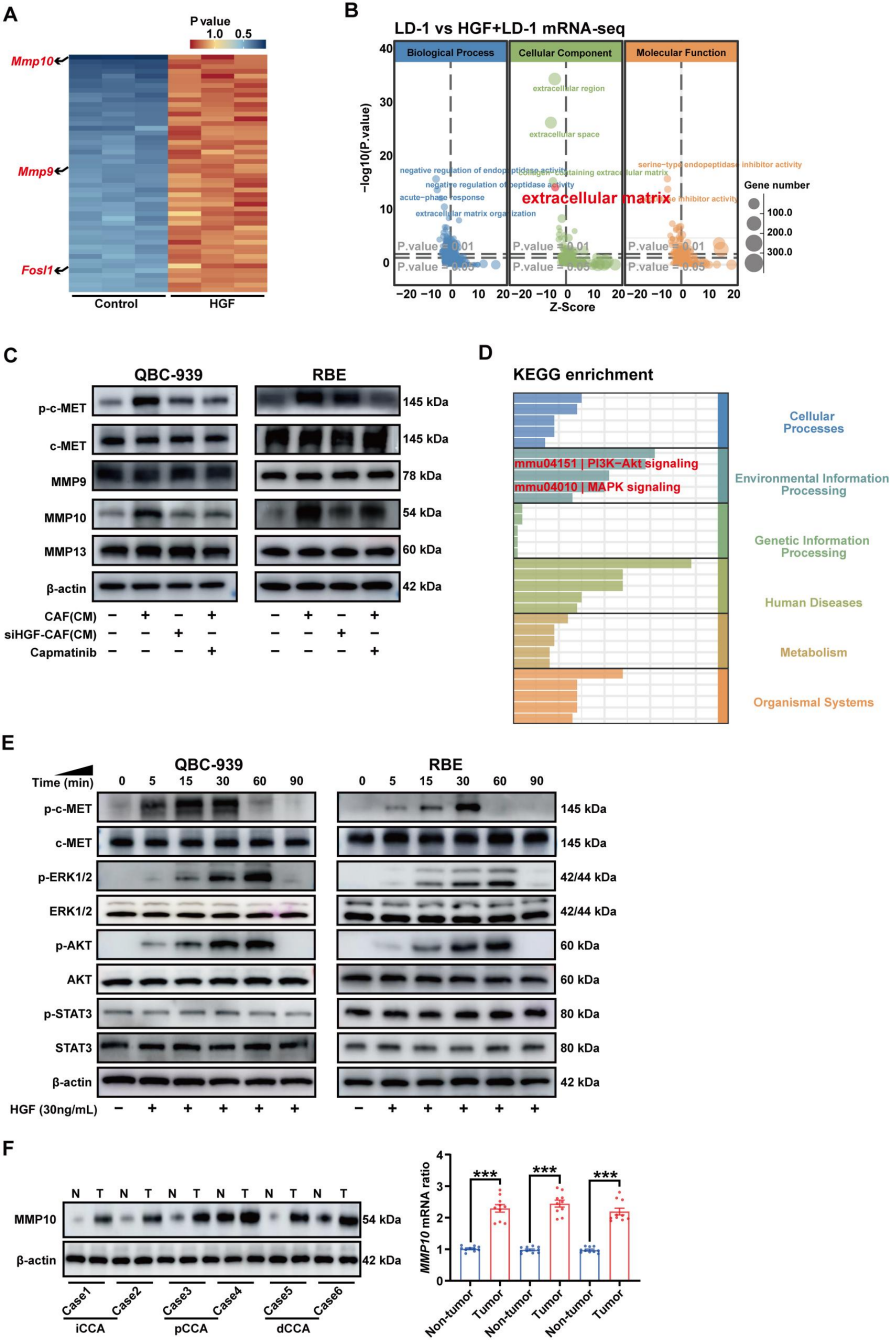
HGF promotes the upregulation of MMP10 expression in CCA cells. (A) Heat map illustrates the significantly differential genes by using mRNA-seq of GFP^+^ LD-1 cells following HGF treatment (TOP 50). (B) Extracellular matrix was significantly enriched in the GO enrichment analysis. (C) Expressions of p-c-MET, MMP9, MMP10, and MMP13 of RBE were detected by using WB following CM treatment from CAFs/siHGF-CAFs and c-MET inhibitor Capmatinib (20 nM). (D) PI3K–Akt and MAPK signaling pathways were significantly enriched in the KEGG enrichment analysis. (E) Phosphorylation of c-MET, ERK, AKT, and STAT3 of CCA cells was detected by using WB after stimulation with 30ng/mL HGF for different times. (F) Expression of MMP10 was detected in iCCA, pCCA, and dCCA tissues, as well as their paratumor tissues, by using WB (left) and qPCR (right). n.s. represents not significant. *, **, and *** represent *P* < 0.05, 0.01, and 0.001 between the indicated groups. Statistical significance was analysed by using paired *t*-test (F, right). Data were from at least three independent experiments and are shown as mean ± SEM.

With mRNA-seq analysis, *Mmp10*, *Mmp9*, and *Fosl1* were significantly upregulated in mice CCA cell line LD-1 following HGF treatment (GSE268676) (Figure 4A and Supplementary Table S4). Based on the gene ontology (GO) enrichment analysis results, the extracellular matrix demonstrates notably increased expression in response to HGF stimulation (Figure 4B and Supplementary Table S5). In addition, MMP10, rather than MMP9 or MMP13, was upregulated following CM treatment from CAFs in CCA cells. However, this effect was blocked by HGF knockdown or c-MET receptor inhibitor Capmatinib (Figure 4C). According to the Kyoto Encyclopedia of Genes and Genomes (KEGG) enrichment analysis and WB, the PI3K-Akt and MAPK signaling pathways exhibited significant upregulation, hinting at a potential link with the invasive and migratory capabilities of CCA cells (Figure 4D and E, and Supplementary Figure S7). By using WB and qPCR, we demonstrated that MMP10 exhibited significantly higher expression levels in three subtypes of CCA tissues compared with paratumoral tissues (Figure 4F).

### FOSL1 mediates the overexpression of MMP10 in CCA cells

**Figure 5.**
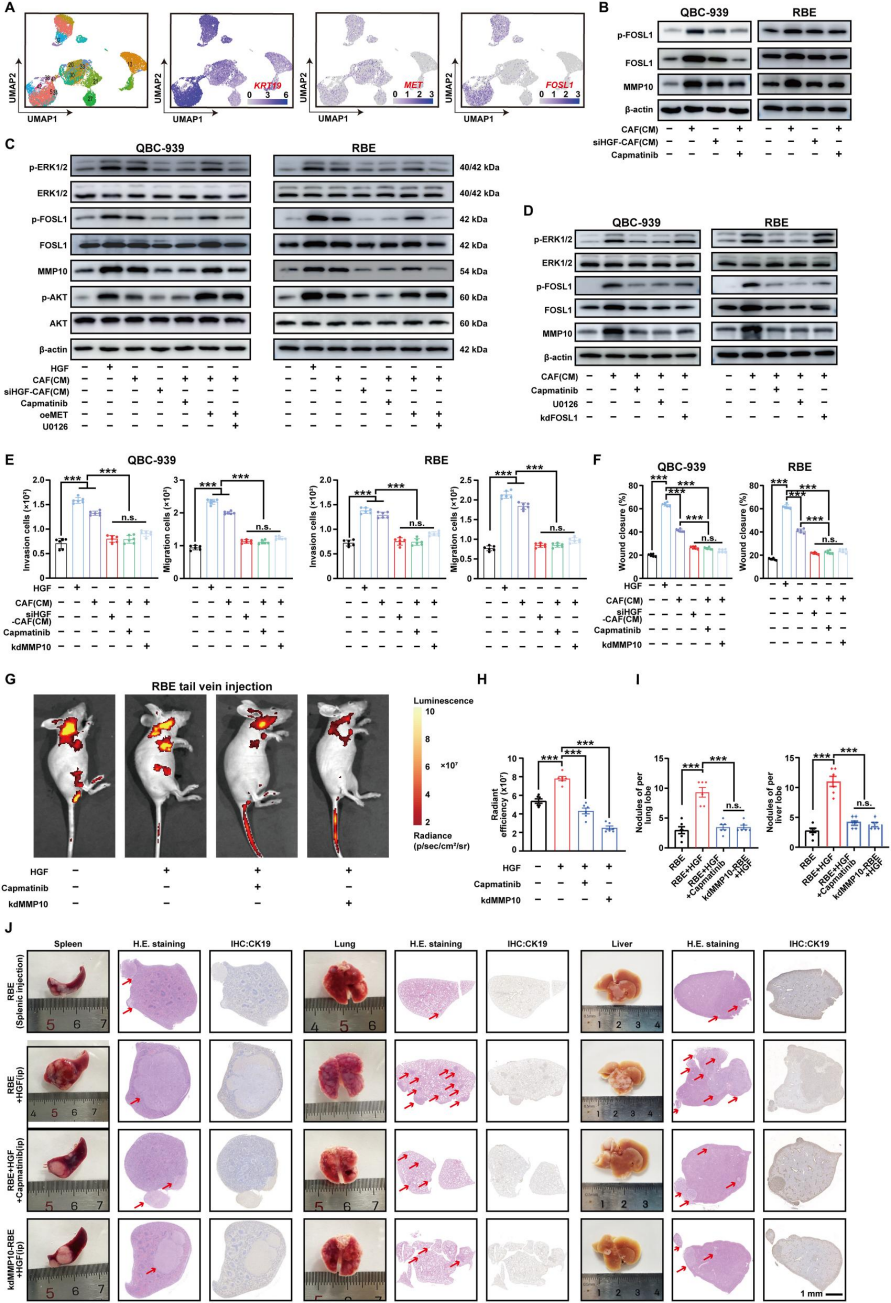
MMP10 mediates the invasion and migration of CCA cells induced by the HGF–c-MET–FOSL1 signaling pathway. (A) In UMAP, *MET* and *FOSL1* were observed to be co-expressed in identical subtypes of tumor epithelial cells. (B) Expressions of p-FOSL1 and MMP10 of QBC-939 and RBE were detected by using WB following CM treatment from CAFs/siHGF-CAFs and Capmatinib (20 nM). (C, D) In the presence/absence of HGF (30 ng/mL), U0126 (20 μM), Capmatinib (20 nM), or MET overexpression plasmid, phosphorylation levels of ERK, FOSL1, and AKT, as well as the expression of MMP10 in CCA cells, were detected by using WB following treatment with CM from CAFs/siHGF-CAFs. (E, F) (MMP10-silenced) CCA cells were incubated in HGF (30 ng/mL), Capmatinib (20 nM), and CM from CAFs or HGF-silenced CAFs. The migration and invasion of QBC-939 (left) and RBE (right) were assessed by using (E) transwell assay and (F) wound-healing assay. (G) After the mice were injected with (MMP10-silenced) RBE via the tail vein, and subsequently HGF (50 ng/kg/day, 7 days) was administered into the abdominal cavity and Capmatinib (30 mg/kg/day, 14 days) was given orally, *in vivo* fluorescence imaging was performed. (H) Radiant efficiency was measured. (I) The number of nodules per lobe in the liver and lung was measured. (J) After immunocompromised mice were splenic injected with (MMP10-silenced) RBE, the liver and lung tissues of the mice were examined. n.s. represents not significant. *, **, and *** represent *P* < 0.05, 0.01, and 0.001 between the indicated groups. Statistical significance was analysed by using one-way ANOVA (E, F, H, and I). Data were from at least three independent experiments and are shown as mean ± SEM.

*Fosl1* exhibited significant upregulation in LD-1 cells following stimulation with HGF (Figure 4A), as well as *MET* and *FOSL1* were observed to be co-expressed in identical subtypes of CCA cells by using our scRNA-seq (Figure 5A). Through WB, we showed that HGF derived from CAFs activated the phosphorylation of FOSL1, ultimately leading to the upregulation of MMP10 (Figure 5B). In addition, c-MET overexpression increased the HGF-mediated phosphorylation of ERK, whereas Capmatinib decreased the c-MET-mediated HGF-induced ERK phosphorylation and ultimately impacted the regulation of p-FOSL1 and MMP10 (Figure 5C and D).

### MMP10 promotes the invasion and migration capabilities of CCA cells

Through transwell experiments and wound-healing assay, we demonstrated that MMP10 mediated HGF derived from CAFs to enhance the invasion and migration abilities of CCA cells (Figure 5E and F, and Supplementary Figures S8 and S9). After the nude mice were injected with RBE, from which MMP10 had been knocked out, via the tail vein, HGF was administered via intraperitoneal injection and Capmatinib was given orally. In addition, we established a splenic injection model of CCA cells in immunocompromised mice. *In vivo* fluorescence imaging and examination of liver and lung tissues from mice injected with CCA cells into the spleen revealed the critical roles of HGF and MMP10 in the distant metastasis process (Figure 5G –J).

### High expression of c-MET and MMP10 showed a positive correlation in clinical cohorts and indicated poor prognosis in CCA

**Figure 6.**
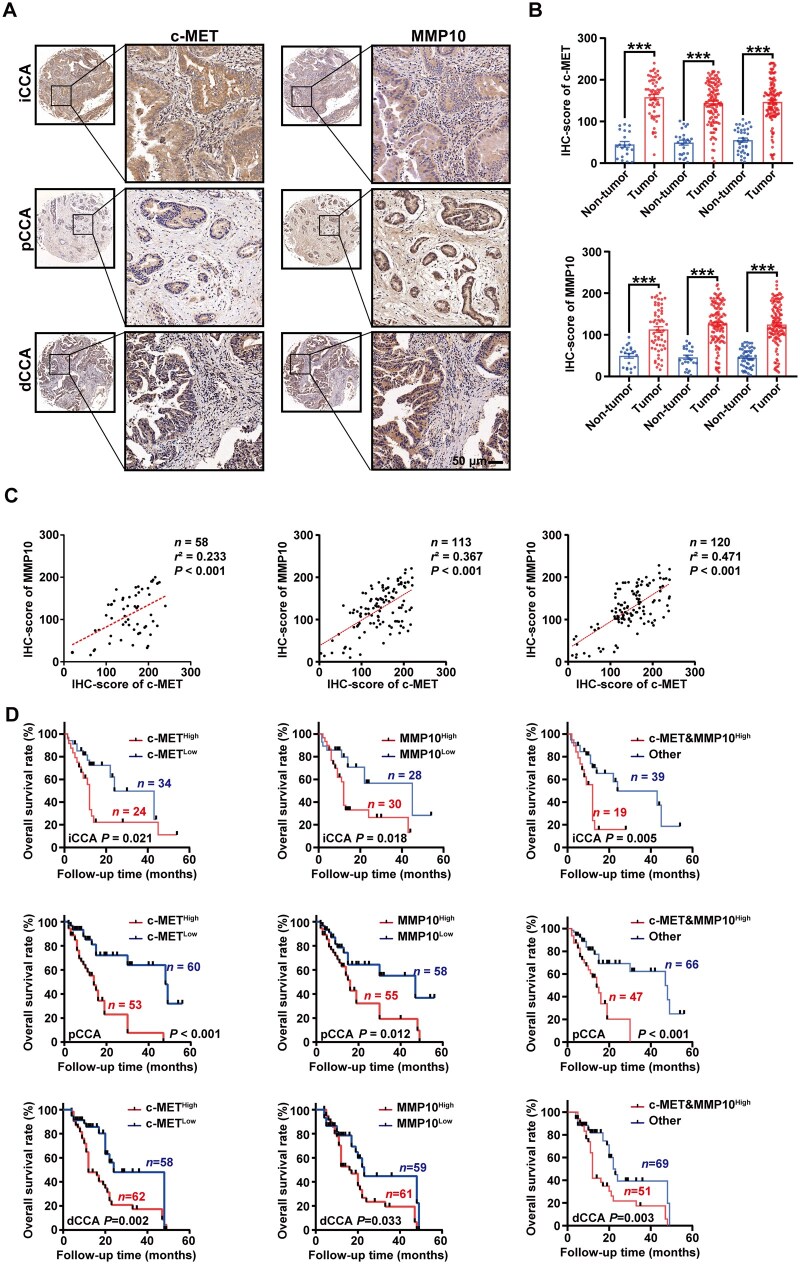
High expression of c-MET and MMP10 showed positive correlation in clinical cohorts and indicated poor prognosis in CCA. (A) Representative images and (B) corresponding IHC scores of c-MET or MMP10 expression in iCCA (*n* = 58), pCCA (*n* = 113), dCCA (*n* = 120), and paratumor tissues. Scale bars: 50 µm. (C) Correlation between c-MET and MMP10 protein expression in iCCA, pCCA, and dCCA was examined. (D) Correlation between the expressions of c-MET or MMP10 and the prognosis of patients with CCA. n.s. represents not significant. *, **, and *** represent *P* < 0.05, 0.01, and 0.001 between the indicated groups. Statistical significance was analysed by using (B) unpaired *t*-test , (C) Pearson test, or (D) log-rank test. Data were from at least three independent experiments and are shown as mean ± SEM.

In IHC results, consistently with previous results, c-MET and MMP10 were mainly co-expressed in CCA cells (Figure 6A). Moreover, the IHC scores of c-MET and MMP10 in CCA were highly positively correlated and significantly upregulated relative to tumor-adjacent tissues (Figure 6B and C). We further investigated the prognostic significance of their expression in our patient cohort. In three subtypes of CCA, high expressions of c-MET and MMP10 were associated with poor prognosis. Additionally, the combined expression of c-MET and MMP10 further exacerbates prognosis, with patients showing dual-positive expression experiencing worse outcomes in CCA (Figure 6D and Supplementary Tables S6–S8).

## Discussion

By using next-generation sequencing and other techniques on CCA tumor tissues and cells, we demonstrated the molecular mechanism by which CAFs in the tumor microenvironment promote the proliferation, invasion, and migration of CCA cells via HGF mediation. In previous studies, we have investigated preliminary research on the function of CAFs, finding that fibroblast growth factor 7 (FGF7) and fibroblast growth factor 19 (FGF19) act as progression-promoting factors influencing the progression of CCA [12, 13]. Furthermore, we detailed the signaling pathways and molecular mechanisms through which MCs remodel the CCA microenvironment [7, 15]. In this study, we have shown that HA released by MCs can activate CAFs, providing a more detailed understanding of the communication network between CAFs and CCA cells.

HGF plays a crucial role in the repair and regeneration of the liver and other tissues [21]. Although research has shown that inflammatory CAFs (iCAFs) can secrete HGF to promote the proliferation of iCCA cells, the initiating factors of CAFs and the precise mechanisms of HGF’s effects remain unclear [8, 22]. As well as previous studies have highlighted the heterogeneity and crosstalk of CAFs to CCA cells [23, 24], our research elucidates the interactions between MCs, CAFs, and CCA cells, with HA and HGF mediating these connections.

In this study, we systematically delineated the precise signaling pathway and the function of HGF in the CCA microenvironment. In CCA, HA promotes CAF proliferation and HGF secretion by activating the HRH2 of CAFs. Here, we provide a panoramic view of the HGF regulatory network in CCA. HGF is a widely distributed growth factor and our findings on its oncogenic function may be applicable not only to CCA, but also to other tumor types. Our results may uncover an aspect of tumor microenvironment regulation involving CAFs and HGF.

**Figure 7.**
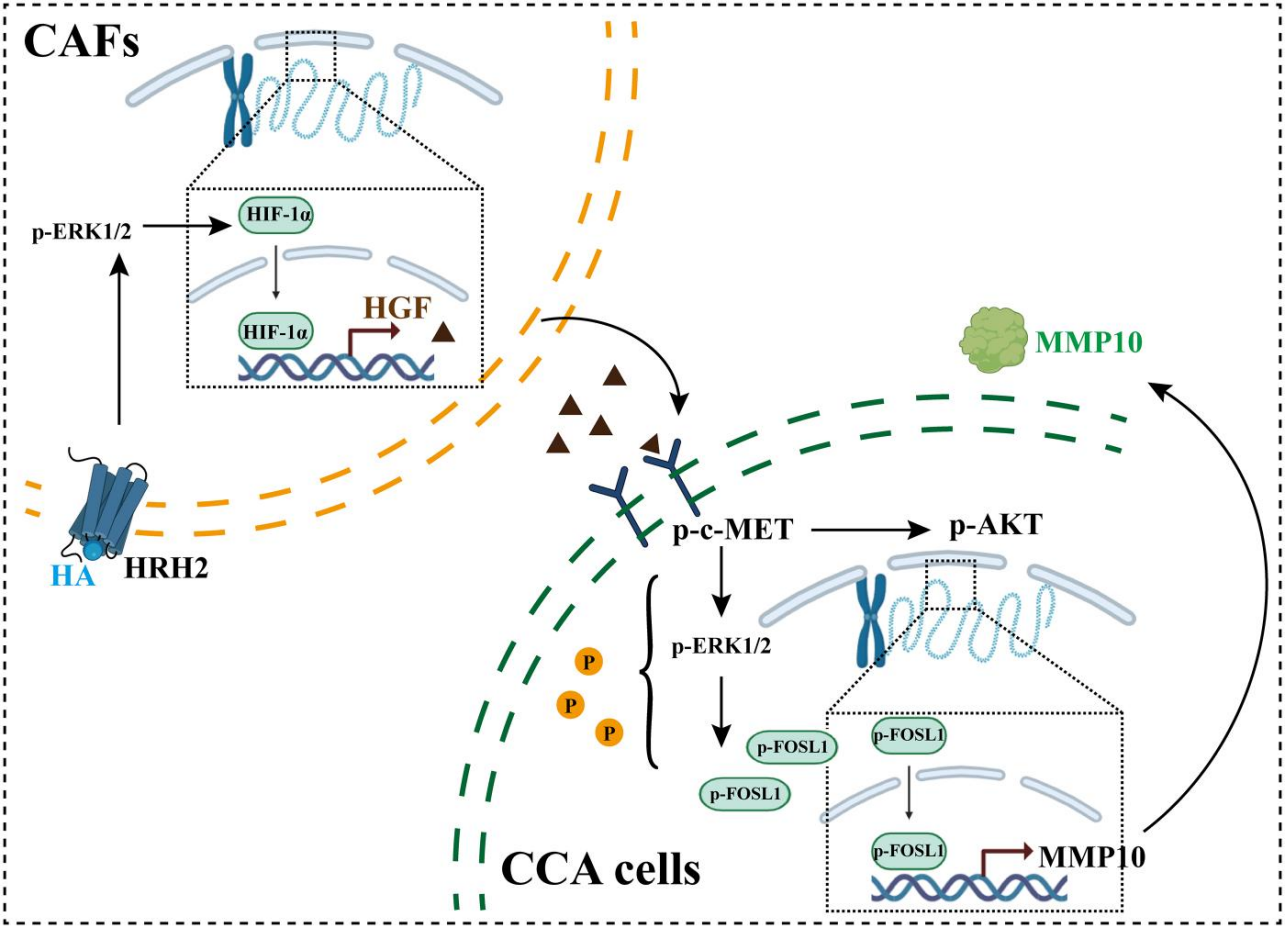
Function of CAFs and HGF in the tumor microenvironment of CCA. In the tumor microenvironment of CCA, HA derived from MCs activates the HA–HRH2 signaling pathway in CAF cells, leading to the phosphorylation of ERK. This activation upregulates the nuclear translocation of transcription factor HIF-1α, which subsequently enhances the transcription, translation, and secretion of HGF. Accumulated HGF binds to the specific receptor c-MET on CCA cells, promoting its phosphorylation. This activation stimulates the MAPK pathway, leading to the upregulation and phosphorylation of the transcription factor FOSL1. Consequently, this leads to the upregulation and secretion of MMP10, thereby promoting the invasion and migration of cholangiocarcinoma cells.

In sum, in the tumor microenvironment of CCA, HA released from MCs activates the HA–HRH2 signaling pathway in CAF cells. This activation increases the nuclear translocation of the transcription factor HIF-1α, which in turn enhances the transcription, translation, and secretion of HGF. The accumulated HGF binds to the specific receptor c-MET on CCA cells, promoting its phosphorylation. This activation stimulates the MAPK pathway, leading to the upregulation and phosphorylation of the transcription factor FOSL1. Consequently, this upregulates and secretes MMP10, thereby promoting the progression of CCA (Figure 7).

Our study identifies HGF as a key effector in CCA progression. Moreover, this study offers new theoretical support for the use of the HA receptor HRH2 inhibitor Cimetidine and the HGF receptor c-MET inhibitor Capmatinib in treating biliary tract tumors. The significance of CAFs and HGF in CCA could lead to the discovery of new potential therapies for treating CCA.

## Supplementary Material

goaf090_Supplementary_Data
