# Supplementary material for: Function of histamine-driven cancer-associated fibroblast and hepatocyte growth factor in the progression of cholangiocarcinoma
Source: Gastroenterol Rep (Oxf). 2025 Oct 1;13:goaf090. doi: 10.1093/gastro/goaf090 (PMC12493034; doi:10.1093/gastro/goaf090)
Supplement: goaf090_Supplementary_Data [file goaf090_supplementary_data.docx]

**Supplementary materials for “Function of histamine-driven cancer-associated fibroblast and hepatocyte growth factor in the progression of cholangiocarcinoma”**

**Xin Wang et al.**

**Supplemental Figure 1**


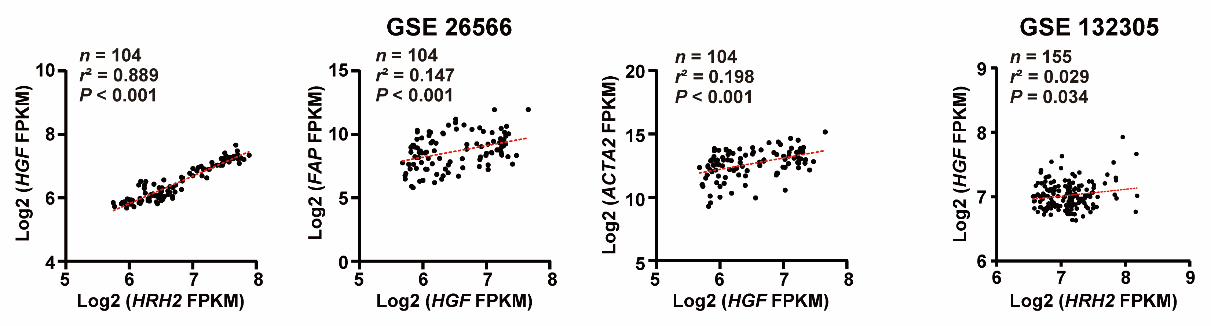


In CCA databases (GSE26566 and GSE132305), a strong correlation between *HRH2* and *HGF* was noted.

**Supplemental Figure 2**


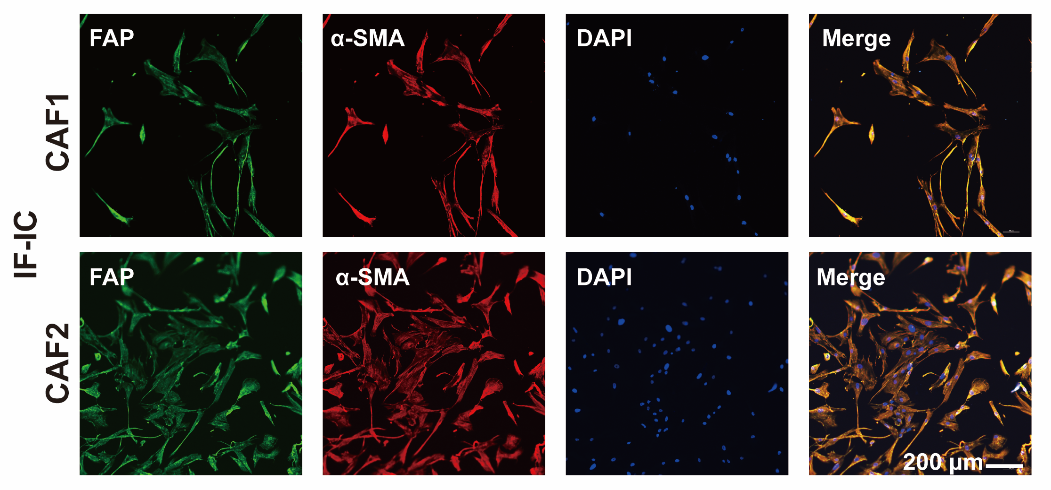


The primary CAFs extracted from CCA tissues were identified by detecting CAF biomarkers (α-SMA and FAP) using IF. Scale bars: 200 µm. Data were from at least 3 independent experiments and shown as mean ± S.E.M.

**Supplemental Figure 3**


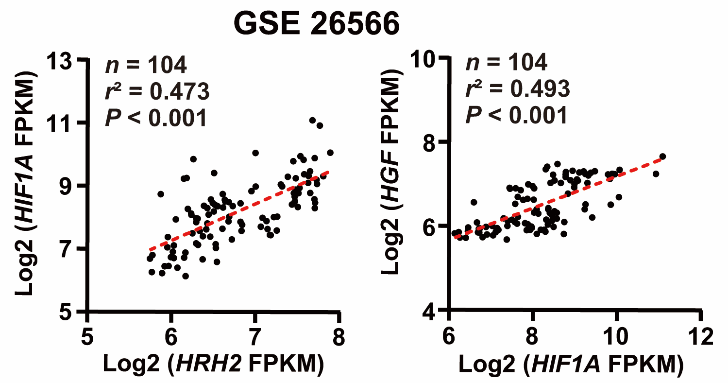


In the CCA database (GSE26566), a robust correlation among *HRH2*, *HIF1A*, and *HGF* was observed.

**Supplemental Figure 4**


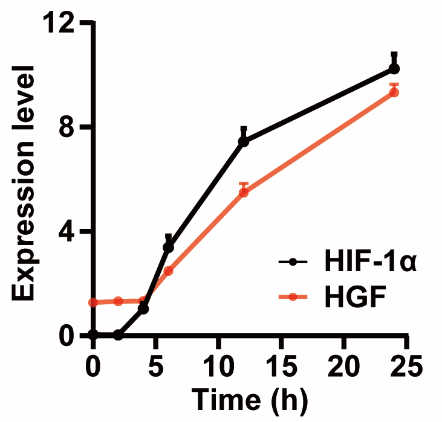


Time-effect correlation between HA (1 μM) stimulation and the expression of HIF-1α and HGF in CAF in Figure 4B was evaluated using WB. Data were from at least 3 independent experiments and shown as mean ± S.E.M.

**Supplemental Figure 5**


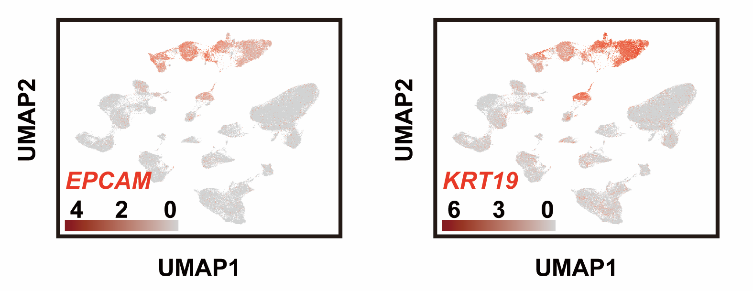


CCA cells were defined by unique biomarker (*EPCAM* and *KRT19*) expressions in UMAP.

**Supplemental Figure 6**


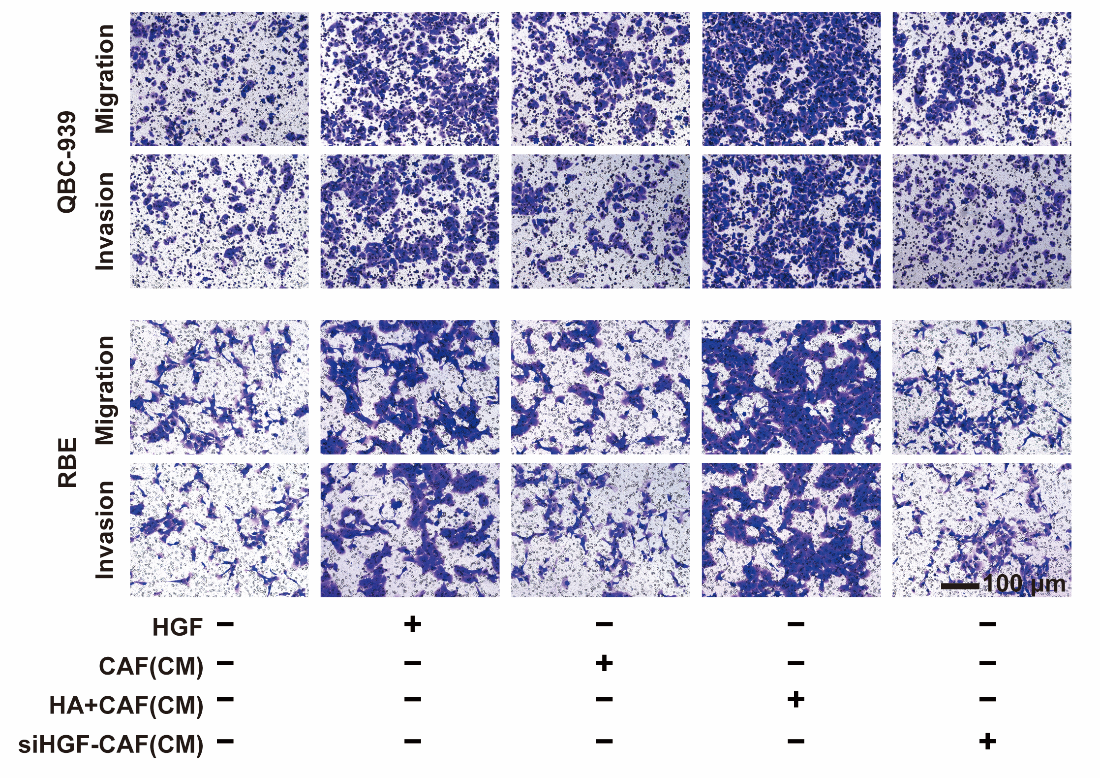


Representative images of transwells assays in Figure 3E. Data were from at least 3 independent experiments and shown as mean ± S.E.M.

**Supplemental Figure 7**


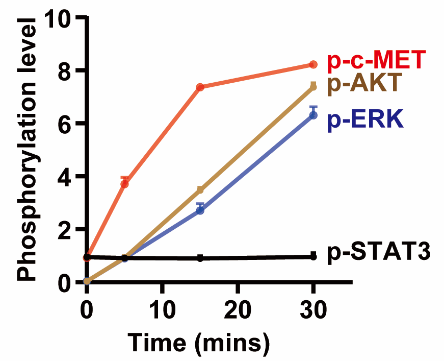


Time–effect correlation between HGF stimulation and phosphorylation of c-MET, AKT, ERK, and STAT3 in QBC-939 in Figure 4E was assessed. Data were from at least 3 independent experiments and shown as mean ± S.E.M.

**Supplemental Figure 8**


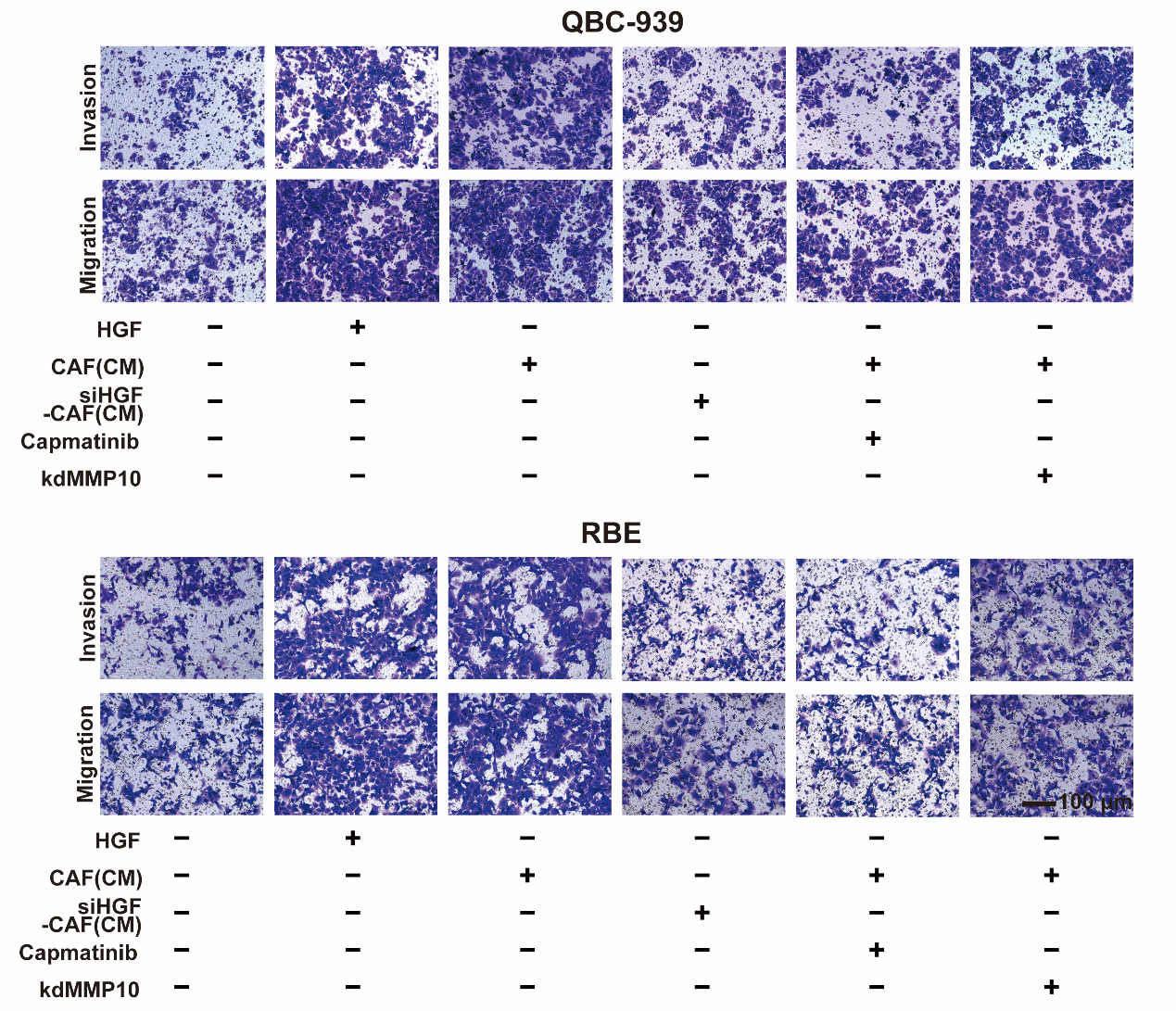


Representative images of transwells assays in Figure 5E. Data were from at least 3 independent experiments and shown as mean ± S.E.M.

**Supplemental Figure 9**


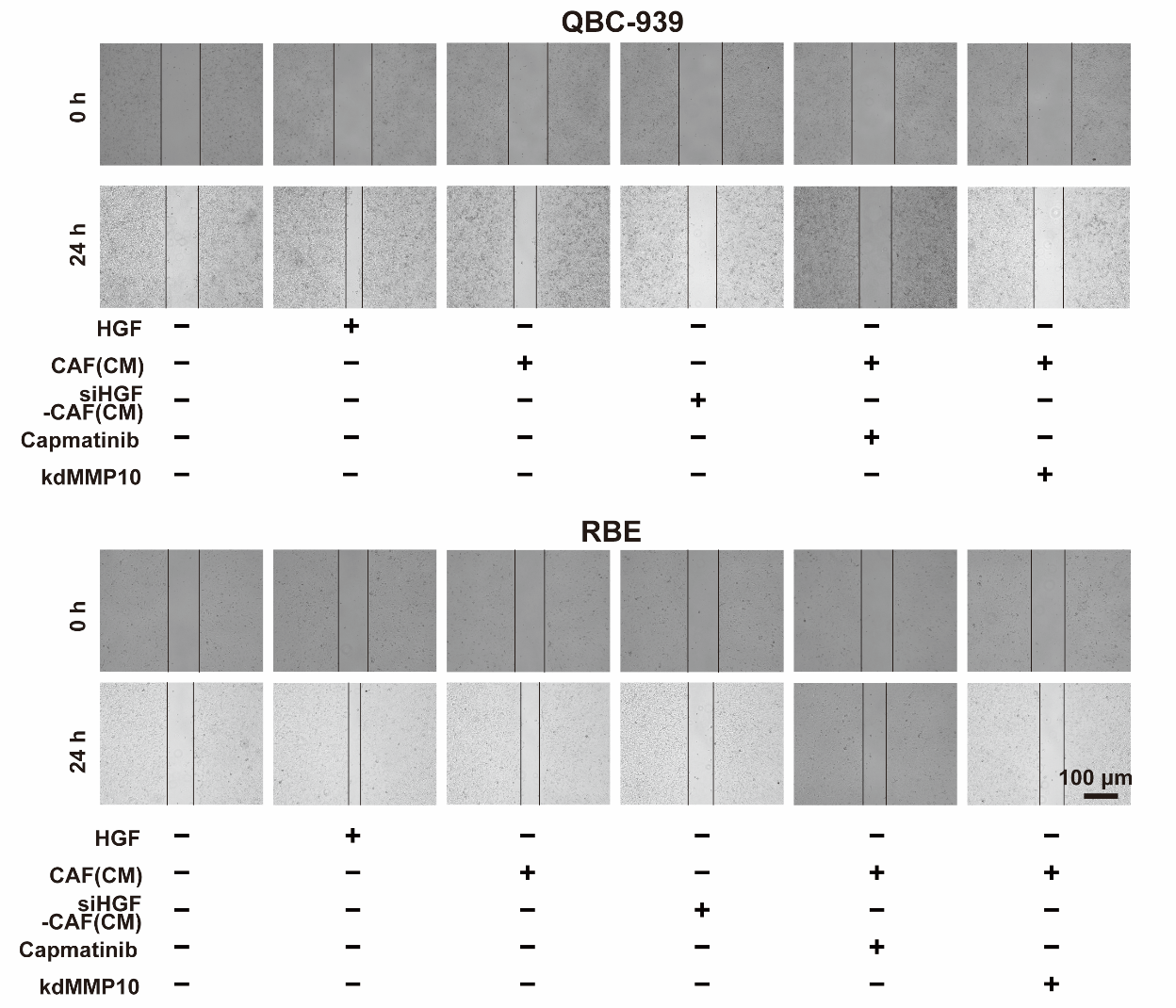


Representative images of wound healing assay in Figure 5F. Data were from at least 3 independent experiments and shown as mean ± S.E.M.

**Supplemental Table 1. List of reagents, antibodies, and ELISA Kits.**

| **Antibodies** | **Source** | **Identifier** |
| --- | --- | --- |
| HRH1 | Immunoway | Cat. No. YT2140 |
| HRH2 | Immunoway | Cat. No. YT2141 |
| HGF | Proteintech | Cat. No.26881-1-AP |
| β-actin | Immunoway | Cat. No.YT0099 |
| HIF-1α | Proteintech | Cat. No.20960-1-AP |
| ERK1/2 | Immunoway | Cat. No.YT1625 |
| p-ERK1/2 | Immunoway | Cat. No.YP0101 |
| c-MET  c-MET(FCM) | Proteintech  Abcam | Cat. No. 25869-1-AP  Cat. No. ab317661 |
| p-c-MET | Immunoway | Cat. No. YP0174 |
| p-c-MET(FCM) | Abcam | Cat. No.ab278553 |
| MMP9 | Immunoway | Cat. No.YT1892 |
| MMP10 | Immunoway | Cat. No.YT2793 |
| MMP13 | Abcam | Cat. No.ab39012 |
| AKT | Immunoway | Cat. No.YT0185 |
| p-AKT | Immunoway | Cat. No. YP0590 |
| STAT3 | Immunoway | Cat. No. YT4443 |
| p-STAT3 | Immunoway | Cat. No. YP0250 |
| FOSL1 | Immunoway | Cat. No. YT1772 |
| p-FOSL1 | Immunoway | Cat. No. YP1344 |
| Histone H3 | Abcam | Cat. No. ab192985 |
| Anti-Hsp60 | Abcam | Cat. No.ab254117 |
| α-SMA | Abcam | Cat. No. ab7817 |
| FAP | Proteintech | Cat. No. 11779-1-AP |
| **Proteins and Chemicals** | **Source** | **Identifier** |
| Histamine | MCE | Cat. No. HY-B1204 |
| Deferoxamine | MCE | Cat. No. HY-B1625 |
| U0126 | MCE | Cat. No. HY-12031A |
| Capmatinib | MCE | Cat. No. HY-13404 |
| HGF | MCE | Cat. No. HY-P70627 |
| **ELISA Kit** | **Source** | **Identifier** |
| Human HGF ELISA Kit | Abcam | Cat. No. ab275901 |

**Supplemental Table 2. List of the primers** [**sequence**](javascript:;) **used for qPCR.**

| **Name** | [**Primer**](javascript:;) [**sequence**](javascript:;) **(5'- 3')** |
| --- | --- |
| *hHRH1* | F: GTGAGGGCAACAAGACCACT  R: GGTTGAGCCCTACTGTGACC |
| *hHRH2* | F: 5′- CCACCATCAGGGAGCACAAA -3′  R: 5′- GCGGTGAAGTAGGGAAACCA -3′ |
| *hHGF* | F: 5′-GCTATCGGGGTAAAGACCTACA-3′  R: 5′-CGTAGCGTACCTCTGGATTGC-3′ |
| *hMET* | F: 5′-CAGCGCGTTGACTTATTCAT-3′  R: 5′-CACTTGTGCAGACTCAGGTTGT-3′ |
| *hGAPDH* | F: 5′-AGAACGGGAAGCTTGTCATCA-3′  R: 5′-GACCTTGCCCACAGCCTTG-3′ |

**Supplemental Table 3. List of the target sequences of siRNAs.**

| **Name** | **The target sequence of siRNAs (5'- 3')** |
| --- | --- |
| scramble | CUCCGAACGUGUCACGU |
| *siHRH1* | GCCUCUUAGAAGACAAGAU |
| *siHRH2* | GGUGAUGAAGUUACAGAAA |
| *siHGF* | CCGCUGGGAGUACUGUGCAAUUAAA |
| *kdMMP10* | GCATCAGGCACCAATTTATTC |
| *kdFOSL1* | CCTCAGCTCATCGCAAGAGTA |

**Supplemental Table 4. Up-regulated mRNAs** **in** **LD-1 stimulated with HGF (Top 50)**

| **Gene** | **A1** | **A2** | **A3** | **B1** | **B2** | **B3** | **Log2FC** | **Pvaule** |
| --- | --- | --- | --- | --- | --- | --- | --- | --- |
| *Mmp10* | 1.68 | 1.83 | 1.58 | 17.12 | 20.44 | 17.47 | 3.4328 | 0.0038 |
| *Serpinb2* | 2.67 | 2.52 | 2.62 | 15.76 | 15.86 | 15.07 | 2.5796 | 0.0002 |
| *Col3a1* | 5.83 | 5.50 | 4.95 | 32.04 | 29.40 | 29.52 | 2.4831 | 0.0005 |
| *S100a4* | 65.37 | 70.06 | 63.52 | 310.55 | 339.49 | 339.60 | 2.3145 | 0.0009 |
| *Ly6g* | 2.73 | 3.79 | 2.27 | 13.08 | 15.26 | 12.40 | 2.2131 | 0.0016 |
| *Prss22* | 3.81 | 4.20 | 3.66 | 16.36 | 17.36 | 17.04 | 2.1215 | 0.0000 |
| *Slpi* | 6.43 | 7.14 | 7.58 | 28.11 | 28.11 | 31.31 | 2.0494 | 0.0010 |
| *Ly6c1* | 30.50 | 31.08 | 30.51 | 120.31 | 129.81 | 126.23 | 2.0310 | 0.0008 |
| *Dynap* | 3.20 | 2.98 | 2.32 | 9.85 | 10.82 | 11.59 | 1.9245 | 0.0008 |
| *Anxa8* | 6.03 | 4.84 | 4.65 | 20.11 | 19.58 | 18.56 | 1.9086 | 0.0000 |
| *Ly6a* | 61.28 | 65.76 | 64.55 | 216.25 | 234.64 | 252.87 | 1.8770 | 0.0034 |
| *Areg* | 15.56 | 15.61 | 14.95 | 54.85 | 54.46 | 58.15 | 1.8605 | 0.0006 |
| *Angpt2* | 1.78 | 2.10 | 1.87 | 7.36 | 6.55 | 6.44 | 1.8258 | 0.0016 |
| *Ccl7* | 4.14 | 4.02 | 3.93 | 13.20 | 13.38 | 16.08 | 1.8193 | 0.0080 |
| *Plet1* | 14.09 | 13.35 | 12.65 | 43.05 | 46.30 | 45.70 | 1.7522 | 0.0002 |
| *Eno3* | 14.48 | 15.22 | 24.21 | 64.91 | 57.78 | 58.80 | 1.7514 | 0.0006 |
| *Nkain1* | 3.53 | 3.47 | 3.66 | 12.68 | 10.88 | 11.05 | 1.6994 | 0.0048 |
| *Mmp19* | 3.20 | 3.60 | 2.66 | 10.92 | 9.88 | 9.72 | 1.6903 | 0.0002 |
| *Krt20* | 1.94 | 1.95 | 1.56 | 5.21 | 5.76 | 5.95 | 1.6347 | 0.0004 |
| *Btc* | 1.78 | 2.02 | 1.63 | 5.61 | 5.88 | 5.31 | 1.6269 | 0.0001 |
| *Nppb* | 2.02 | 2.68 | 2.17 | 7.66 | 6.18 | 7.15 | 1.6130 | 0.0030 |
| *Serpine1* | 19.30 | 18.88 | 17.46 | 60.50 | 56.16 | 53.14 | 1.6096 | 0.0019 |
| *Vcam1* | 3.07 | 3.35 | 2.72 | 8.83 | 9.16 | 9.50 | 1.5875 | 0.0000 |
| *Mmp9* | 2.12 | 2.04 | 2.03 | 6.32 | 6.52 | 5.78 | 1.5869 | 0.0024 |
| *Itgb7* | 44.94 | 41.76 | 41.30 | 130.29 | 128.69 | 120.13 | 1.5666 | 0.0004 |
| *Sele* | 3.31 | 2.98 | 3.10 | 8.28 | 9.47 | 9.41 | 1.5336 | 0.0027 |
| *Hao1* | 3.86 | 3.25 | 3.55 | 9.65 | 10.08 | 11.06 | 1.5304 | 0.0012 |
| *Ptgs2* | 6.77 | 6.86 | 6.48 | 17.82 | 18.65 | 21.52 | 1.5281 | 0.0073 |
| *Lipg* | 3.07 | 2.96 | 2.36 | 8.22 | 8.32 | 7.62 | 1.5250 | 0.0001 |
| *Serpinb9b* | 3.22 | 2.84 | 2.64 | 7.16 | 8.25 | 9.00 | 1.4894 | 0.0060 |
| *Ssc5d* | 3.11 | 2.86 | 3.20 | 8.26 | 8.38 | 9.07 | 1.4877 | 0.0006 |
| *Col1a1* | 4.98 | 5.15 | 4.26 | 12.16 | 12.45 | 15.25 | 1.4710 | 0.0092 |
| *3300005D01Rik* | 9.17 | 9.03 | 7.64 | 23.32 | 24.46 | 23.15 | 1.4571 | 0.0000 |
| *Ifitm1* | 32.84 | 36.99 | 32.37 | 76.98 | 99.09 | 103.59 | 1.4523 | 0.0164 |
| *Aqp3* | 2.31 | 2.97 | 1.98 | 6.26 | 6.58 | 6.85 | 1.4403 | 0.0009 |
| *Spp1* | 117.34 | 126.84 | 101.63 | 276.30 | 318.57 | 334.67 | 1.4265 | 0.0031 |
| *Rab3b* | 6.46 | 6.29 | 6.89 | 18.27 | 18.61 | 15.76 | 1.4216 | 0.0052 |
| *Fn1* | 46.53 | 44.56 | 41.20 | 123.53 | 116.54 | 113.36 | 1.4178 | 0.0002 |
| *Serpine2* | 1.63 | 1.64 | 1.55 | 4.38 | 4.19 | 4.30 | 1.4162 | 0.0000 |
| *Add2* | 1.60 | 1.59 | 1.65 | 4.84 | 4.15 | 3.86 | 1.4086 | 0.0114 |
| *Ocstamp* | 3.17 | 3.04 | 2.50 | 7.59 | 7.68 | 7.57 | 1.3923 | 0.0015 |
| *Sgk2* | 1.86 | 1.90 | 1.59 | 4.83 | 4.50 | 4.69 | 1.3884 | 0.0000 |
| *Ramp3* | 2.87 | 3.70 | 3.32 | 9.14 | 8.65 | 7.89 | 1.3774 | 0.0006 |
| *Krt19* | 65.67 | 63.70 | 61.08 | 170.93 | 164.84 | 158.67 | 1.3764 | 0.0003 |
| *Fosl1* | 22.49 | 21.41 | 22.60 | 46.23 | 61.92 | 63.85 | 1.3708 | 0.0238 |
| *Ifi27l2a* | 41.41 | 45.77 | 49.95 | 112.09 | 117.49 | 116.90 | 1.3373 | 0.0001 |
| *Eps8* | 3.09 | 3.15 | 2.63 | 7.47 | 7.52 | 7.39 | 1.3366 | 0.0008 |
| *Mtmr11* | 8.46 | 8.28 | 7.92 | 19.65 | 21.26 | 21.08 | 1.3298 | 0.0007 |
| *Pcolce2* | 15.21 | 14.44 | 13.35 | 37.92 | 36.07 | 33.93 | 1.3276 | 0.0006 |
| *Ano1* | 10.74 | 10.28 | 10.30 | 26.85 | 25.44 | 25.83 | 1.3191 | 0.0002 |

A1-–A3 = LD-1 cultured in DMEM without HGF (30 ng/mL);

B1-–B3 = LD-1cultured in DMEM with HGF (30 ng/mL).

**Supplem****ental Table 5.** **GO Terms in QBC 939 stimulated with HGF (TOP10)**

|  | **GO_Term** | **GO_Category** | **Rich.Factor** | **P.value** | **Q.value** |
| --- | --- | --- | --- | --- | --- |
| GO:0005576 | Extracellular region | Cellular Component | 0.1 | **** | **** |
| GO:0005615 | Extracellular space | Cellular Component | 0.0939 | **** | **** |
| GO:0004867 | Serine-type endopeptidase inhibitor activity | Molecular Function | 0.25 | **** | **** |
| GO:0010951 | Negative regulation of endopeptidase activity | Biological Process | 0.2034 | **** | **** |
| GO:0062023 | Collagen-containing extracellular matrix | Cellular Component | 0.1565 | **** | **** |
| GO:0031012 | Extracellular matrix | Cellular Component | 0.1614 | **** | **** |
| GO:0030414 | Peptidase inhibitor activity | Molecular Function | 0.2314 | **** | **** |
| GO:0010466 | Negative regulation of peptidase activity | Biological Process | 0.2295 | **** | **** |
| GO:0006953 | Acute-phase response | Biological Process | 0.4412 | **** | **** |
| GO:0030198 | Extracellular matrix organization | Biological Process | 0.1728 | **** | **** |

**Supplemental Table 6. The prognostic significance of clinicopathological characteristics in iCCA.**

| **Characteristics** | | **3-year OS** | ***P*^a^** | **HR** | **95%CI** | ***p*^b^** |
| --- | --- | --- | --- | --- | --- | --- |
| **Age (years)** | < 65 | 25.9 |  |  |  |  |
|  | ≥ 65 | / | 0.340 |  |  |  |
| **Gender** | female | / |  |  |  |  |
|  | male | 30.9 | 0.857 |  |  |  |
| **Tumor size** | < 2.5cm | 17.2 |  |  |  |  |
|  | ≥ 2.5cm | 40.4 | 0.956 |  |  |  |
| **Differentiation** | Well/Moderate | 26.7 |  |  |  |  |
|  | Poor | / | 0.853 |  |  |  |
| **T stage** | T1 + T2 | 18.7 |  |  |  |  |
|  | T3 + T4 | / | 0.350 |  |  |  |
| **N stage** | N0 | 20.4 |  |  |  |  |
|  | N1 + N2 | / | 0.653 |  |  |  |
| **M stage** | M0 | 27.8 |  |  |  |  |
|  | M1 | / | 0.231 |  |  |  |
| **TNM stage** | I + II | 22.1 |  |  |  |  |
|  | III + IV | / | 0.878 |  |  |  |
| **MET** | Low | 24.8 |  | 1 |  |  |
|  | High | 11.1 | **0.021** | 1.988 | 0.90-4.37 | 0.089 |
| **MMP10** | Low | 28.3 |  | 1 |  |  |
|  | High | 13.2 | **0.018** | 2.283 | 0.94-5.53 | 0.067 |
| **MET&MMP10** | Other | 37.3 |  | 1 |  |  |
|  | Both high | / | **0.005** | 0.951 | 0.15-6.17 | 0.958 |

**Abbreviations:**

OS = overall survival,

HR = hazard ratio;

CI = confidence interval;

^a^ Calculated by log-rank test.

^b^ Calculated by Cox-regression Hazard model

**Supplemental Table 7. The prognostic significance of clinicopathological characteristics in pCCA.**

| **Characteristics** | | **3-year OS** | ***P*^a^** | **HR** | **95%CI** | ***p*^b^** |
| --- | --- | --- | --- | --- | --- | --- |
| **Age (years)** | < 65 | 22.0 |  |  |  |  |
|  | ≥ 65 | 34.2 | 0.191 |  |  |  |
| **Gender** | female | 49.7 |  |  |  |  |
|  | male | 15.6 | 0.202 |  |  |  |
| **Tumor size** | < 2.5 cm | 25.6 |  |  |  |  |
|  | ≥ 2.5 cm | / | 0.091 |  |  |  |
| **Differentiation** | Well/Moderate | 30.7 |  | 1 |  |  |
|  | Poor | / | **0.018** | 1.896 | 0.96-3.76 | 0.067 |
| **T stage** | T1 + T2 | 24.2 |  |  |  |  |
|  | T3 + T4 | 18.0 | 0.614 |  |  |  |
| **N stage** | N0 | 28.3 |  |  |  |  |
|  | N1 + N2 | 30.5 | 0.811 |  |  |  |
| **M stage** | M0 | 28.6 |  |  |  |  |
|  | M1 | / | 0.523 |  |  |  |
| **TNM stage** | I + II | 24.1 |  |  |  |  |
|  | III + IV | 30.7 | 0.928 |  |  |  |
| **MET** | Low | 48.0 |  | 1 |  |  |
|  | High | 7.6 | **<0.001** | 4.089 | 2.03-8.22 | **<0.001** |
| **MMP10** | Low | 36.8 |  | 1 |  |  |
|  | High | 9.6 | **0.012** | 0.498 | 0.10-2.40 | 0.385 |
| **MET&MMP10** | Other | 49.8 |  | 1 |  |  |
|  | Both high | / | **<0.001** | 2.131 | 0.30-15.03 | 0.448 |

**Abbreviations:**

OS = overall survival,

HR = hazard ratio;

CI = confidence interval;

^a^ Calculated by log-rank test.

^b^ Calculated by Cox-regression Hazard model

**Supplemental Table 8. The prognostic significance of clinicopathological characteristics in dCCA.**

| **Characteristics** | | **3-year OS** | ***P*^a^** | **HR** | **95%CI** | ***p*^b^** |
| --- | --- | --- | --- | --- | --- | --- |
| **Age (years)** | < 65 | 14.5 |  |  |  |  |
|  | ≥ 65 | 19.4 | 0.738 |  |  |  |
| **Gender** | female | / |  |  |  |  |
|  | male | 12.3 | 0.420 |  |  |  |
| **Tumor size** | < 2.5 cm | 13.4 |  |  |  |  |
|  | ≥ 2.5 cm | 21.0 | 0.747 |  |  |  |
| **Differentiation** | Well/Moderate | 16.8 |  |  |  |  |
|  | Poor | / | 0.940 |  |  |  |
| **T stage** | T1 + T2 | / |  |  |  |  |
|  | T3 + T4 | 20.1 | 0.421 |  |  |  |
| **N stage** | N0 | 12.4 |  | 1 |  |  |
|  | N1 + N2 | / | **0.042** | 0.537 | 0.25-1.15 | 0.109 |
| **M stage** | M0 | 17.0 |  |  |  |  |
|  | M1 | / | 0.332 |  |  |  |
| **TNM stage** | I + II | / |  |  |  |  |
|  | III + IV | 26.9 | 0.501 |  |  |  |
| **MET** | Low | 48.0 |  | 1 |  |  |
|  | High | 8.7 | **0.002** | 2.447 | 1.33-4.52 | **0.004** |
| **MMP10** | Low | 22.4 |  | 1 |  |  |
|  | High | 6.5 | **0.033** | 0.988 | 0.31-3.20 | 0.984 |
| **MET&MMP10** | Other | 19.6 |  | 1 |  |  |
|  | Both high | 5.8 | **0.003** | 1.310 | 0.32-5.45 | 0.710 |

**Abbreviations:**

OS = overall survival,

HR = hazard ratio;

CI = confidence interval;

^a^ Calculated by log-rank test.

^b^ Calculated by Cox-regression Hazard model
